# Towards an Optimal Pressure Tap Design for Fluid-Flow Characterisation at Microscales

**DOI:** 10.3390/ma12071086

**Published:** 2019-04-02

**Authors:** Tomás Rodrigues, Francisco J. Galindo-Rosales, Laura Campo-Deaño

**Affiliations:** 1 CEFT, Departamento de Engenharia Mecânica, Faculdade de Engenharia da Universidade do Porto, Rua Dr. Roberto Frias, 4200-465 Porto, Portugal; tomas.rodrigues@fe.up.pt; 2 CEFT, Departamento de Engenharia Química, Faculdade de Engenharia da Universidade do Porto, Rua Dr. Roberto Frias, 4200-465 Porto, Portugal; galindo@fe.up.pt or frosales@inegi.up.pt; 3 Instituto de Ciência e Inovação em Engenharia Mecânica e Engenharia Industrial, Rua Dr. Roberto Frias, 400, 4200-465 Porto, Portugal

**Keywords:** microchannels, microfluidics, pressure drop, pressure taps

## Abstract

Measuring fluid pressure in microchannels is difficult and constitutes a challenge to even the most experienced of experimentalists. Currently, to the best of the authors’ knowledge, no optimal solution are being used for the design of pressure taps, nor guidelines concerning their shape and its relation with the accuracy of the readings. In an attempt to address this issue, a parametric study was devised to evaluate the performance of different pressure tap designs, 18 in total. These were obtained by combining three shape parameters: sub-channel width (*w*) and sub-channel–tap radius (*R*) or angle (α), while having the sub-channel length kept constant. For each configuration, pressure drop measurements were carried out along several lengths of a straight microfluidic rectangular channel and later compared to an analytical solution. The microchannels were fabricated out of PDMS using standard soft-lithography techniques, pressure drop was measured with differential pressure sensors, the test fluid was DI water and the flow conditions varied from creeping flow up to $Re_{c}$∼100. Pressure taps, having smooth contours (characterised by the radius *R*) and a sub-channel width (*w*) of 108μm, performed the best with results from that of radius R=50μm only falling short of the theory by a mere ∼5%.

## 1. Introduction

Microfluidics is the science and technology that deals with systems that process small amounts of fluid, using geometries with dimensions of tens to hundreds of microns [1]. However, the important length-scale in microfluidics is not the overall size of the device but rather the length-scale that determines flow behaviour. In fact, the main advantage of microfluidics is using scaling laws and continuum breakdown for investigating new effects at this scale [2], since certain fundamental differences can be observed between the behaviour of fluids moving in large channels and those flowing through microscale channels [3].

The ability to control and analyse the flow of fluids in microfluidic devices is important for developing tools in Lab-on-a-Chip (LOC) systems [1,4,5,6,7,8,9,10,11,12,13]. Pressure drop measurements within microfluidic channels can greatly aid in designing passive microfluidic pumps [6] and obtaining rheological properties in microfluidic rheometers [1,4,8,9,10,12,13], for example. Yet, determining the pressure inside a microfluidic channel is not as straightforward as one might think. For instance, a viable solution for measuring the pressure drop caused by the presence of a microbot inside a blood vessel-like microchannel, which would add new information to the study of the dynamic efficiency of these devices [14], is still lacking [15,16].

The most common pressure sensors used in microfluidics are capacitive [17] and piezoresistive [18,19,20]. Besides these, other variations, such as optical [21,22,23], vision-based [24] and resonant sensors, do exist. A general review of several pressure-sensing technologies for microdevices can be found in Eaton and Smith [25]. These days, external piezoresistive pressure transducers with high preciseness and sensitivity are the ones most used. These are highly accurate, can read both absolute and relative pressure in a wide measuring range and connect to pressure taps in the channels, just like their macroscale counterparts.

Besides the commercially available external pressure sensors, several other methods for measuring pressure in microchannels have been reported. The base material from which the microchannels are made, for instance, thanks to its intrinsic properties, might allow for specific measuring methods. That is the case of silicon-based microchannels, a rather decaying solution for microdevice fabrication thanks to the much cheaper polydimethylsiloxane (PDMS) alternative. Several research groups [22,26,27] have used silicon-based microchannels mainly because silicon can be routinely etched and therefore several sensing elements can be incorporated. The process of pressure measuring within silicon-based microdevices follows a different principle as that of PDMS-based ones. It involves reflecting an imposed laser beam upon a deflecting silicon channel wall and relating the reflection angle to the channel pressure [22]. When it comes to PDMS channels, some researchers [7,28,29,30,31,32] have used the elastic nature of the material to their advantage, relating the deflection of the inner walls to the pressure in the channel by means of imaging tools. However, this method and others alike often require additional fabrication steps [29] in order to incorporate extra channel layers into the main PDMS channels [7,28,30,31], fluorescent particles [7,32,33], or even introduce separate probing fluids [28,30].

Banerjee and Mastrangelo [34] set out to develop a pressure-sensing system for microfluidic devices based on low-leakage microballoons. These compressible microballoons change their size in response to pressure changes. The applicability of this method is determined by the size of the channel in which its implementation is desired, mainly due to the diameter of the microballoons (12–15μm).

Park et al. [20] developed a carbon fibre-based piezoresistive pressure sensor. While traditional piezoresistive sensors have four diffused silicon wire sensing piezoresistors in a closed Wheatstone bridge configuration, in the one here developed these piezoresistors are replaced with carbon fibres. Compared to silicon wire, carbon fibres are easier to fabricate and have higher gauge factor.

Another carbon fibre-based piezoresistive pressure sensor was developed by Lee and Choi [19], this time with a PDMS diaphragm instead of the conventional silicon one. Here, not only do the piezoresistors get replaced by carbon fibres but also the diaphragm is different. With a PDMS diaphragm larger deformations under low pressure are possible, since its Young modulus is greater than that of silicon. The PDMS diaphragm was 50μm-thick and the carbon fibres were manufactured from polyacrylonitrile (PAN).

Also based on PDMS deformation, Tsai and Kaneko [24] proposed an on-chip pressure sensor that requires no additional instrument nor electricity. Instead, the pressure is related to the colour intensity that a coloured fluid displays by flowing in and out of a sensing chamber due to its deformation.

Kohl et al. [22] carried out pressure measurements by an optical membrane-based method, in order to determine friction factors.

Following up on their previous work, a microfluidic platform with internal pressure measurements was described by Kohl et al. [23]. In this platform, the deformation of silicon membranes with pressure is read through an optical laser-based process. These deformations are then converted to pressure. The silicon membranes are electronics-free, so no piezoresistors or capacitors are required. As explained earlier, internal measuring systems often require extra fabrication steps in order to be incorporated into microdevices and this case is no exception.

Lei et al. [17] developed a flexible capacitive pressure sensor for plantar applications. It consists of a PDMS dielectric layer, with the sensing electrodes attached, inside a flexible printed circuit film substrate.

A novel conductive gel-based pressure sensor was developed by Li et al. [18]. This gel is made of PDMS and either carbon or metal particles. The sensor proved to be capable of measuring within the typical pressure range of most microfluidic devices. In line with other similar systems however, it requires a dedicated fabrication process in order to be implemented.

In this work the most practical solution for pressure drop measuring will be used, external pressure sensors. This is the simplest way for measuring pressure drop in existing PDMS microdevices with minimal modifications, hence being the most straightforward, repeatable and flexible method of all [35]. In addition to only requiring the external sensors, with no design or extra device requirements, these can easily be coupled with high-speed data acquisition (DAQ) hardware, allowing for real-time dynamic measurements.

Despite the need for precise pressure measurements in microfluidics, the number of studies in this field is limited [36]. Mostly because pressure measuring at this scale is not particularly easy. Several problems have been reported, a great number of those associated with pressure tap design issues. For instance, one of the most frequent is the housing of air bubbles inside the taps or in the sub-channels that lead to them. These affect the accuracy of the readings tremendously, significantly distorting the measurements. Surface tension phenomena are also quite common, caused mainly by the high surface area–volume ratios characteristic of microfluidic devices. Problems like these are frequently related to poorly designed pressure-sensing structures with long and narrow sub-channels, which promote such undesired effects.

This study aims to minimise such difficulties when measuring pressure in microchannels, by developing guidelines for the design of well-functioning pressure taps and ultimately proposing an optimal tap configuration.

## 2. Materials and Methods

### 2.1. Microdevice Design

**Figure 1:**
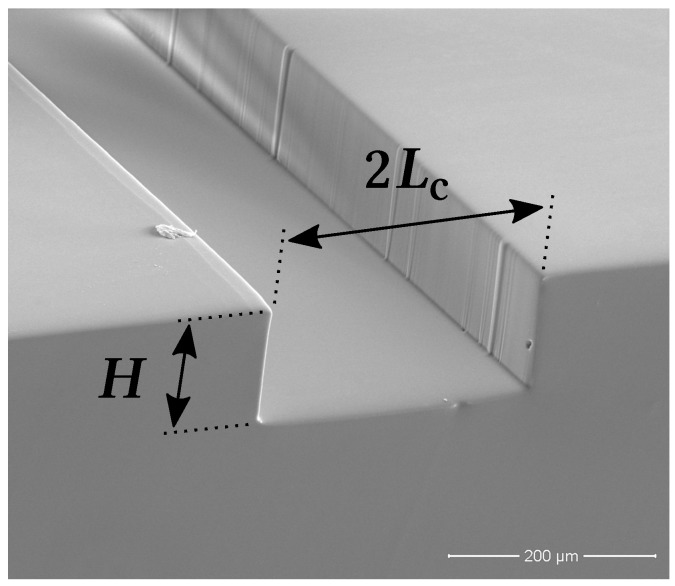
Scanning electron microscopy (SEM) image of the microchannel 270 μm ×100μm cross-section: $2L_{c}$ is the channel width and *H* is the channel height, $L_{c}$ being its characteristic length-scale.

The experimental setup consists of a straight 10mm long rectangular microfluidic channel. The rectangular cross-section is 270μm wide ($2L_{c}$) and 100μm tall (*H*), setting the channel characteristic length-scale, $L_{c}$, at 135μm (see Figure 1). Next to the channel inlet and outlet ports there are two built-in pressure taps (both ∼1.5mm in diameter) for assessing the pressure drop along the entire length of the microchannel.

**Figure 2:**
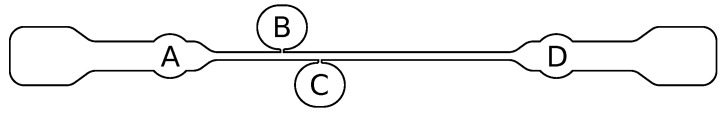
Pressure taps A, B, C and D location along the microchannel.

Moreover, a couple of static pressure taps were added to the main flow channel. These taps are ∼1mm away from each other and on opposite sides of the straight channel. They were placed far enough from the inlet so that the flow would be fully developed by the time it reached them, avoiding any entry effects. Specifically, ∼7mm away from the start of the 10mm long straight section (much longer than the conventional minimum entrance length of $10\;\times \;2L_{c}$). There are, therefore, a total of four different pressure-sensing zones in the microchannel—A, B, C and D (see Figure 2).

Note that the later taps (B and C) do not have a built-in configuration like the ones first mentioned (A and D). These, however, branch out from the main channel via a narrower sub-channel that connects to a roundish area (also ∼1.5mm in diameter) in which the static pressure can be sensed. The configuration of these taps was the subject of our parametric study, where several designs were tested and evaluated both qualitatively and quantitatively. That is, evaluation by experimental observation of their performance, such as the propensity for air bubble housing, and by pressure data analysis and comparison to theory.

A wide range of configurations was obtained by combining three shape parameters: sub-channel width (*w*), and sub-channel–tap radius (*R*) or angle (α). The sub-channel length was kept at a constant 50μm throughout all designs. Up to three values were considered per parameter, as shown in Table 1. This means that a total of 18 different configurations can be arranged with these variables. Note that *w* can only combine with either *R* or α at a time.

By comparing the results obtained by the different pressure tap designs, the influence of the three shape parameters considered in the accuracy of the whole pressure measuring process can be assessed. This way, an optimal tap configuration (among the ones tested) can be ultimately proposed.

### 2.2. Microdevice Fabrication

From the conceptualisation and design of the microchannels to their materialisation, stands a two-stage fabrication process in between. First, with the designs that had been developed, SU-8 molds of the microchannels were fabricated. In order to make these, a hard mask of the channels was created to begin with. This consisted on an aluminium (Al) coated (a 200nm film) Corning^®^ glass substrate cut to size, in which the microchannels were cut out. With this, optical lithography (or, as it is often referred to, photolithography) was used to create the channels master molds on SU-8 coated silicon substrates. Once the photo-resist molds were prepared, the microchannels could then be fabricated, using standard soft-lithography techniques [37]. This marks the start of the second stage of the fabrication process. The channels were created using a two-part Sylgard^®^ 184 PDMS polymer kit, mixed to a weight ratio of 10:1 (pre-polymer:cross-linker). The two parts were mixed and allowed to degas under vacuum in a desiccator, so that the air bubbles introduced by the mixing action would be removed. A silanization (in gas) treatment, with trimethylchlorosilane (TMCS), was applied to the molds surface in order to make it hydrophobic ($\mathrm{contact}\mathrm{angle}>90^{\circ }$) and avoid peeling issues upon demolding. The PDMS mixture was then poured onto the silicon master molds and cured in an oven to accelerate the cross-linkage process. At $80{\;}^{\circ }C$, a 30min baking time frame is enough for achieving a soft PDMS consistency that will allow for easy manipulation and piercing, once cooled down. When fully cured, the PDMS layers were cut and peeled off from the molds. These were then pierced and bonded to PDMS spin coated glass microscope slides, to close off the channels. The resulting PDMS chips were baked one last time for half an hour and left to rest overnight, before being ready for testing. The average accuracy of dimensions is lower than 5μm for the microchannels.

### 2.3. Pressure Drop Measurements

All tests were carried out with a Newtonian fluid, de-ionised (DI) water ($∼998\;\mathrm{kg}\;m^{-3}$, ∼0.001Pas) at a controlled room temperature of $∼20{\;}^{\circ }C$. The Reynolds number—ratio of inertial to viscous forces—characterising the flow in the microchannel is:

(1) $$Re_{c}=\frac{\rho U_{m}D_{h}}{\mu }$$

where ρ is the working fluid density, μ is its dynamic viscosity, $U_{m}$ is the mean velocity in the channel and $D_{h}=2\times 2L_{c}H$/$\left(2L_{c}+H\right)$ is its hydraulic diameter. The flow rates, *Q*, considered ranged from $0.0185\;\mu L\;s^{-1}$ to $18.5\;\mu L\;s^{-1}$, which leads to a $Re_{c}$ interval from ∼0.1 up to ∼100. The mean velocities, $U_{m}=Q/A_{T}$, in the microchannel were $0.686\;\mathrm{mm}\;s^{-1}\leq U_{m}\leq 686\;\mathrm{mm}\;s^{-1}$, where $A_{T}$ is the channel cross-section area. At this length-scale the transition from laminar to turbulent flow occurs for $Re_{\mathrm{cr}}\geq 200$ [38], hence the flow regimens here considered are strictly laminar.

Flow was driven through the device using a neMESYS low pressure syringe pump (Cetoni GmbH) with a 14:1 gear ratio. The pump controlled the microchannel inlet flow rate while the outlet was left open to the atmosphere, to balance the flow. Depending on the required flow rate different glass syringes (Hamilton^®^ Gastight) were used, ensuring ‘pulsation free’ dosing. These were connected to the channel using flexible Tygon^®^ tubing and stainless steel precision dispensing tips (Nordson Corporation, Westlake, OH, USA).

**Figure A1:**
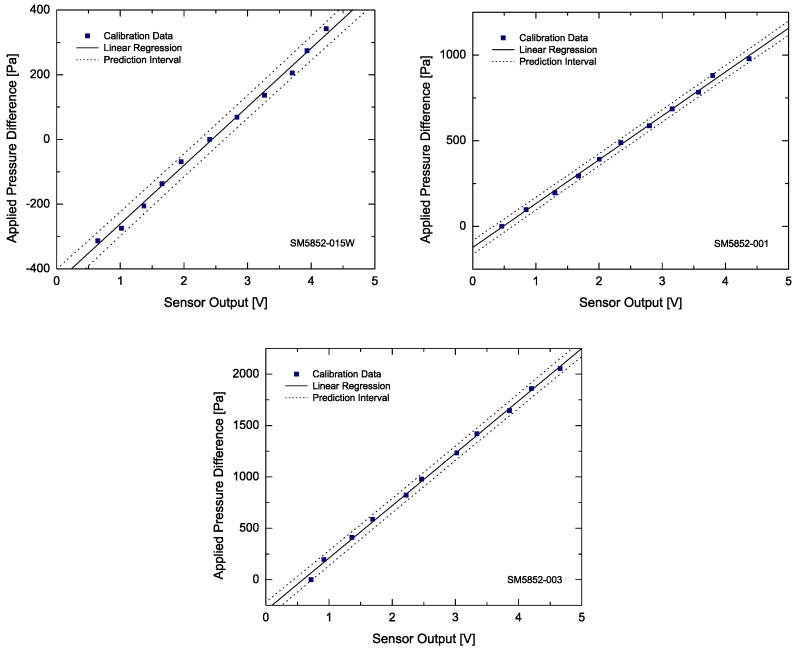
Calibration curves of the differential pressure sensors (a detailed description of the process can be found in Section 2.3). The prediction interval is for a confidence level of 95%.

The pressure drop, ΔP, at different flow rates was measured using Silicon Microstructures, Inc., Milpitas, CA, USA. SM5852 Series piezoresistive differential pressure transducers, with a response time of 2ms and a differential pressure range of ±2.1kPa altogether. The sensors were calibrated by applying known hydrostatic height differences (ΔP=ρgΔh, where *g* is gravity and Δh is the applied height difference) to both ends and measuring the corresponding voltage outputs, correlating the two afterwards. The calibration curves obtained were later used to relate the measured voltage output of the sensors to a differential pressure drop reading, at different applied flow rates (these are shown in Figure A1 of Appendix A). Depending on the flow rates tested, pressure sensors with different ranges/sensitivities were employed. A 5V DC power supply was used to power the pressure sensors, which were connected to a computer via a National Instruments DAQ card in order to record the output data (at an acquisition rate of 1000Hz) using a custom built LabVIEW program. For each flow rate tested, the transient response of the transducers was continuously recorded until a steady-state condition (i.e., steady flow) was achieved when the voltage signal levelled off. Such state is characterised by a plateau with superimposed low amplitude oscillations, mostly caused by electronic noise. The pressure data was then sampled at a rate of 10Hz for never less than 60s (this was always at least 600 data points). An arithmetic averaging method was performed over the collected data.

**Figure 3:**
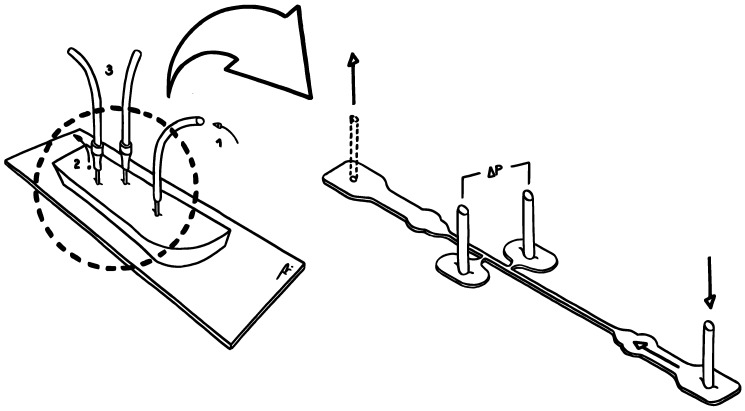
Sketch of the experimental setup (microfluidicchip+microchannel): (1) Inlet. (2) Outlet—left open to the atmosphere to balance the flow. (3) Tubing that establishes the connection to the pressure sensor ports.

The connections between the transducers ports and the pressure taps were also done via Tygon^®^ tubing and stainless steel connectors. In order to avoid excessively long transients in the pressure drop, upon changing the flow rate, the length of the tubing was reduced as much as possible [6]. These transient periods are likely to be caused by air compressibility and component compliance effects. While the flow is running and the pressure is building up, the fluid is not directly touching the sensor die (or membrane). Instead, a column of air is trapped inside the tubing between these. Therefore, the pressure that is read is equivalent to the pressure undergone by the air, since this one is compressed to equal the pressure in the taps. Along with the compression, a pressure gradient is generated, leading to believe that the amount of time required for the liquid to compress the air to steady-state is partially responsible for the sensor transient response. The elasticity of the various components of the setup also impacts this lag. Due to their deformability, both the tubing and the PDMS expand under pressure, receding later to an undeformed state when steady-state is reached. Despite the air compressibility effects which are inevitable, the use of shorter tubing helps to minimise transient response times, as mentioned. All things considered, the resolution of the dynamic pressure readings was limited by the sampling rate of the DAQ card, response time of the pressure sensors and signal-noise from vibrations and flexing of elastic components. Figure 3 shows a sketch of the experimental setup.

## 3. Results

Having the channel four pressure-sensing zones—A, B, C and D (recall Figure 2)—it was possible to measure the pressure drop along four different sections of its length: AB, BC, CD and AD. The length of each of these sections is listed in Table 2, from which one concludes that $\Delta P_{\mathrm{BC}}<\Delta P_{\mathrm{AB}}<\Delta P_{\mathrm{CD}}<\Delta P_{\mathrm{AD}}$.

According to conventional laminar flow theory [39], for fully developed, steady-state Newtonian flow in a straight rectilinear rigid channel of known dimensions, corresponding to section BC, the pressure drop is given by the force balance $wd\Delta P=2L\left(w+d\right)\tau$, which derives from the Navier-Stokes equation and where *L* is the channel length, *w* and *d* are the channel width and depth, respectively, and τ is wall shear stress. From this balance yields $\Delta P=2\tau L\left(w+d\right)/wd∼2\tau L/d$, which shows the influence of channel aspect ratio (w/d) on the pressure drop. For L/d≫1 and w/d≫1, the following expression can be computed:

(2) $$\frac{\Delta P_{\mathrm{analyt}}}{L}=\frac{12Q\left(1+\frac{d}{w}\right)\mu }{wd^{3}}\;,$$

with *Q* being the flow rate and μ the dynamic viscosity. A similar solution to the Navier-Stokes equation, using Fourier sum representation (within 10% error), can be found in Bruus [40].

**Figure 4:**
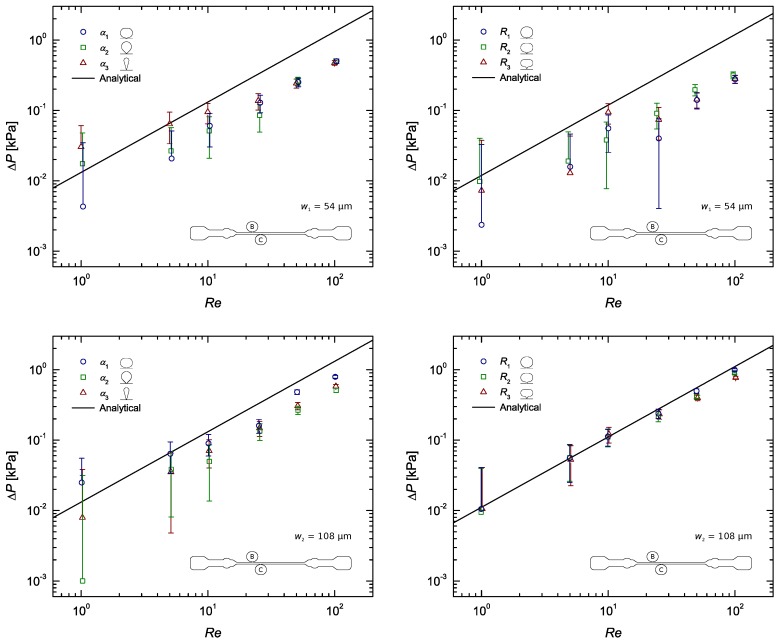

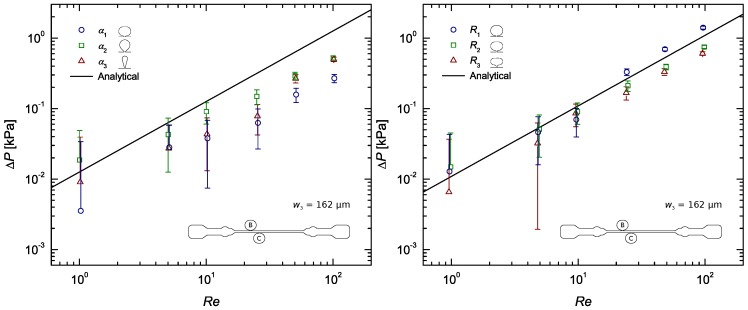
Steady-state pressure drop measurements as a function of the Reynolds number for the flow of DI water along a rectangular microchannel (section BC), compared with the analytical solution (Equation (2)): Pressure taps with sub-channel widths $w_{1}$, $w_{2}$ and $w_{3}$; α taps on the left and *R* taps on the right.

**Figure A2:**
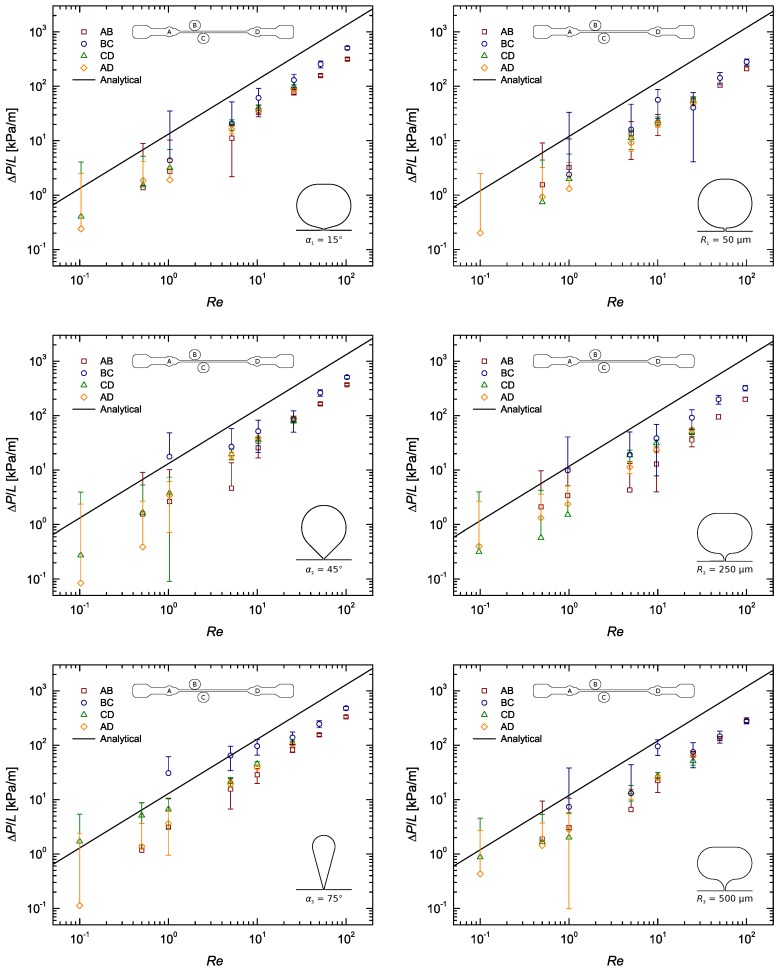
Pressure gradient as a function of the Reynolds number for the flow of DI water along a rectangular microchannel: Pressure taps with sub-channel width $w_{1}=54\;\mu m$; α taps on the left and *R* taps on the right.

**Figure A3:**
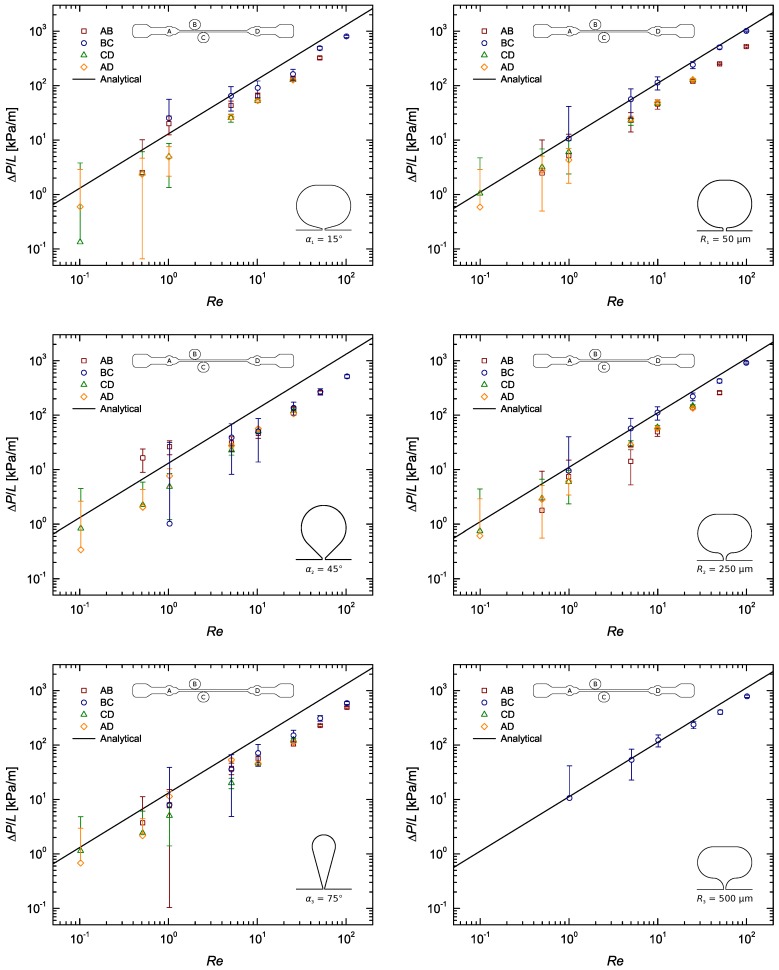
Pressure gradient as a function of the Reynolds number for the flow of DI water along a rectangular microchannel: Pressure taps with sub-channel width $w_{2}=108\;\mu m$; α taps on the left and *R* taps on the right (the lack of data in the bottom right panel was due to leakage problems).

**Figure A4:**
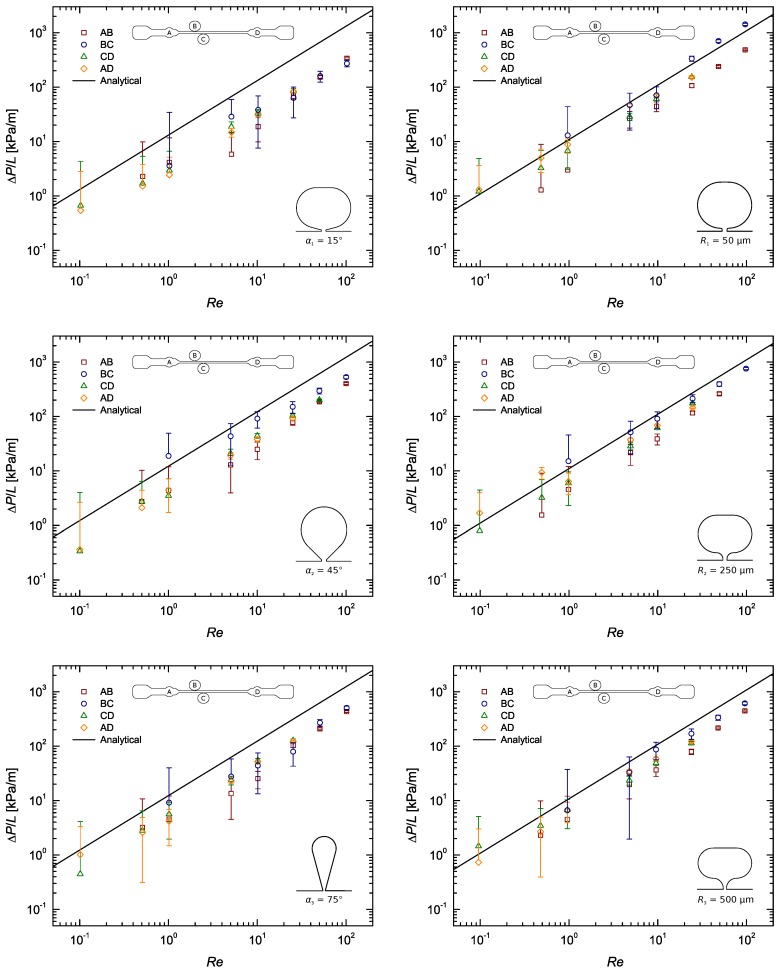
Pressure gradient as a function of the Reynolds number for the flow of DI water along a rectangular microchannel: Pressure taps with sub-channel width $w_{3}=162\;\mu m$; α taps on the left and *R* taps on the right.

The experimental results are presented in a more condensed manner in Figure 4, where only the data of section BC was considered. For the sake of clarity, results comparing the measurements corresponding to the four pressure sensing zones (AB, BC, CD and AD) are included in the Appendix A. Figure A2, Figure A3, Figure A4 show the normalised static pressure drop, ΔP/L, versus Reynolds number for the flow of DI water along sections AB, BC, CD and AD of the microchannel with all pressure taps of sub-channel width $w_{1}$, $w_{2}$ and $w_{3}$. Here, pressure drop per unit length, ΔP/L, was plotted to rule out the influence of the different section lengths on the results. Error bars contemplate experimental uncertainties in the sampled voltage (randomness in the readings), DAQ card (signal acquisition and analog–digital conversion errors), pressure sensors (accuracy- and sensitivity-related errors) and calibration process (linear fitting error).

## 4. Discussion

As expected, the pressure gradient increased linearly with Re. Some minor non-linearity might have been caused by the compliance of the PDMS walls: as pressure increases the walls may deform slightly, increasing the inner dimensions of the channel, and by consequence, a change in the microchannel cross-section leads to a change in pressure drop as well. Note that the analytical profile (Equation (2)) assumes rigid channel walls, not contemplating the elastic nature of PDMS nor the possibility for the cross-section to deform. Some works have discussed the importance of bulk deformation in rectangular microchannels. Gervais et al. [5] studied the elastic deformation of PDMS microchannels under imposed flow rates. Following up on this study, Cheung et al. [35] proposed an elastic rectangular expression capable of predicting the pressure drop in deformable PDMS microchannels. Results obtained by Gervais et al. [5] and Cheung et al. [35] showed significant non-linearity between the experimental pressure drop data and the theoretical predictions based on conventional rigid channel theory at large flow rates and under high pressure drops. That is because in a bulged microchannel the pressure drop no longer changes linearly with flow rate [41]. However, the reported discrepancies between theoretical and experimental data seen at large flow rates are much more pronounced under high pressure drops and in channels with high aspect ratios. In these cases, the slope of the pressure drop versus flow rate curves decreases significantly as *Q* increases. In fact, Cheung et al. [35] reported that at low pressures (<5kPa) the experimental data remained linear. Our microfluidic channel is characterised by a low–moderate aspect ratio of ∼2.7 and pressure drops never exceed ∼2.1kPa, hence it was expected that channel deformation would not have a significant impact on the results. In line with the predictions, the experimental results in Figure 4 seem to properly follow the linear behaviour of the theoretical profile, with a few punctual exceptions (the reason for those will be addressed further below). Thus, whilst explaining some minor deviations off the linear trend, one can conclude that the deformability of PDMS has little impact on the results.

**Figure 5:**
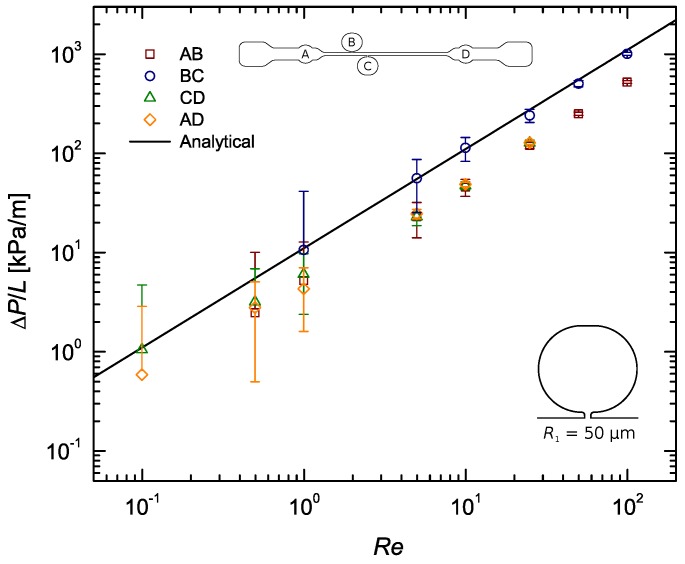
Pressure gradient as a function of the Reynolds number for the flow of DI water along a rectangular microchannel: Pressure tap with sub-channel width $w_{2}=108\;\mu m$ and sub-channel–tap radius $R_{1}=50\;\mu m$.

The pressure sensors were statically calibrated, however the measured pressure drops did not agree with the computed ones most of the times, falling systematically below the theoretically predicted curve. A closer look at Figure A2, Figure A3, Figure A4 show that this systematic behaviour is more pronounced in the results from sections AB, CD and AD, with the data from section BC being sometimes in good agreement with the theory. Take Figure 5, for example. This difference, regarding distances AB, CD and AD, is believed to be mainly caused by the built-in configuration of taps A and D. Their roundish built-in design inherently adds expansion/contraction zones which alter the flow behaviour dramatically as it passes through theses sections. Consequently, pressures A and D are measured outside of the straight channel, immediately before and after an increase (contraction) and relief (expansion) in pressure, respectively, whereas pressures B and C actually correspond to those felt inside the straight channel. In addition to that, tap A is placed just after the channel inlet, where the flow is nowhere near developed. All this, naturally, has an effect on the pressure drop measured along distances AB, CD and AD, which may be the main reason behind the disparity observed between results from these sections and those from section BC, consistently closer to the theoretical profile. The configuration of pressure taps A and D is also believed to have influence on the scattering of the data observed for each Re. As explained in Section 3, by plotting ΔP/L it was expected that the data for each Re would all be coincident. Figure A2, Figure A3, Figure A4 show that these are more often than not apart. This happens because no measurements were made under exactly the same conditions. While the data from section BC can be said to be the most ‘clean’, being located where the flow is fully developed, the data from the other sections all share a common error source. The expansion/contraction introduced by the configuration of taps A and D affect both the upstream and downstream pressure signals in section AD, whilst in section AB only the upstream signal is affected (A) and in section CD the same thing happens only for the downstream signal (D), and while these differences are true, one can go even further and conclude that the pressure drop measured in sections AB, CD and AD is not representative of the actual pressure difference felt inside the straight channel along those very same lengths, precisely due to the described errors. Therefore, it would be impossible for the ΔP/L results measured along these sections to match those measured along section BC.

Another effect that adds to this one, although only relevant to the results from AB and CD, is hole pressure error. This error though, is strictly associated with pressure taps B and C since these are connected to the main flow channel by a sub-channel each. Hole pressure, $P_{H}=P_{w}-P_{s}$, is the difference between the actual pressure at the channel walls, $P_{w}$ (i.e., the pressure that a flush-mounted sensor would measure) and the pressure measured by a recessed sensor, $P_{s}$, just like ours. This difference is due to the normal stresses in the fluid, which cause streamlines to bend when passing over pressure holes. The tension in the streamlines causes a tensile stress to be exerted over the holes, making the sensors measure a lower pressure than the actual pressure in the bulk flow. Fortunately, the magnitude of this error is consistent with the size of the hole, being therefore reasonable to assume that its influence on the results can be neglected when compared to the influence of the expansion/contraction introduced by taps A and D. It is worth mentioning that for differential pressure measurements this effect cancels itself out, as explained by Scott [42], meaning that in the measurements carried out along section BC there is no such error.

Yet, this does not explain the gap often found between experimental and theoretical results to its full extent. A closer look at Figure A2, Figure A3, Figure A4 show that the results from section BC sometimes meet their theoretical prediction. While those from the remaining sections (AB, CD and AD) often fall short of it. This happens precisely for the reasons explained above, regarding the expansion/contraction introduced by the configuration of pressure taps A and D, which led to the conclusion that the only section that is able to provide somehow ‘clean’ readings is BC. Moreover, the theoretical profile considered is only valid for plain straight channels, not contemplating the contraction/expansion effects seen in sections AB, CD and AD.

Despite BC being the best section to draw conclusions from, it also fails to meet the theory for several cases, which can only be interpreted as having been caused by either poor pressure tap design (the focus of this work), external effects (i.e., effects whose control falls beyond the experimentalist reach and that cannot be suppressed), or a combination of both. Having said that, it is therefore crucial to address first and foremost the external effects that were likely to have caused the deviations from the theoretical profile, since these are applicable to all the measurements carried out. The effects that are believed to have been the cause for this behaviour are presented herein.

Air bubbles trapped in the microchannels are though to have had the most impact in the errant pressure measurements. The presence of air inside microchannels is known to be capable of distorting pressure readings [11,42], either by blocking the flow or by ‘absorbing’ some of this pressure due to its compressibility, in a process very similar to that of the bulging of PDMS channels under pressure. Air bubbles were mostly observed inside the pressure taps and near the inlet/outlet of the channel. The more or less likelihood for air bubbles to get stuck inside pressure taps B and C was seen as a consequence of the shape of these taps.

Other considerations worth mentioning are those of viscosity, entrance length and 3D effects. Studies have shown that water retains its bulk viscosity value to within ∼10% even in films as thin as 5nm [43]. Therefore, its viscosity in the wall region is not expected to vary significantly from that of the bulk, discarding the influence of this parameter on the results. Regarding the entrance length, the placement of pressure taps B and C was careful not to be in a region where the flow would still be in development. Hence, no extra pressure loss due to entry effects needs to be considered. Naturally, having a rectangular cross-section creates 3D flows inside the microchannel, yet it is believed that the level of confinement (ratio of the channel height to the channel width), $\Lambda =H/2L_{c}$∼0.4, is not high enough to enhance these effects to such a degree that they would have impact on the results [44].

Now that the external effects which likely lead to the errant pressure measurements have been characterised, remarks on the best and worst performing pressure tap configurations can be made. First though, in order to get a sense of the true scale of the deviations observed (between the experimental and theoretical data) these must be quantified. As so, Table 3, Table 4 and Table 5 show the underlying percentage error (PE) in the results, $\left|\Delta P_{\mathrm{analyt}}-\Delta P_{\exp}\right|/\Delta P_{\mathrm{analyt}}$, for selected Re and per section.

From all the results displayed in this section, the following conclusions (based mainly on the data collected from section BC) about pressure tap design can be drawn.

Immediately, a difference between the results from $w_{1}$ taps and those from $w_{2}$ and $w_{3}$ taps stands out. All $w_{1}$ taps show worst results than the rest, most likely due to the surface tension, here enhanced by the sub-channel high surface area–volume ratio. This gets clearer by taking a closer look at Figure 4. Hence, one can conclude that sub-channel widths of w≤54μm do not produce accurate results, being significantly affected by interfacial phenomena.

Regarding the remaining configurations (all $w_{2}$ and $w_{3}$ taps) another major distinction can be made, this time between the results obtained with α taps and those obtained with *R* taps. It seems that α taps perform worst, regardless of the *w*. The explanation for this might lie in the fact that the shape contour of the *R* taps is rather smooth, whereas the one of the α taps is not. That said, it is therefore reasonable to conclude that assuring a smooth shape with no edges is a good practice when designing a pressure tap.

Taking a closer look at Table 4 and Table 5, for $w_{2}$ and $w_{3}$ taps respectively, and comparing the *R* taps with 108μm ($w_{2}$) and 162μm ($w_{3}$) sub-channel width, it becomes evident that the results obtained with the narrower sub-channel are better. So much better in fact, that with this sub-channel, of width $w_{2}$, the experimental results were found to often match the theoretical profile. This happened particularly at the lower Reynolds numbers, Re<50 (see Figure 4), sometimes only failing theory by ∼2%. The reasons for the difference observed between the results with $w_{2}$ and $w_{3}$ taps are not entirely clear yet. The mild deviation from the theoretical profile at high Re was most likely caused by the slight compliance of the channels inner PDMS walls.

**Figure 6:**
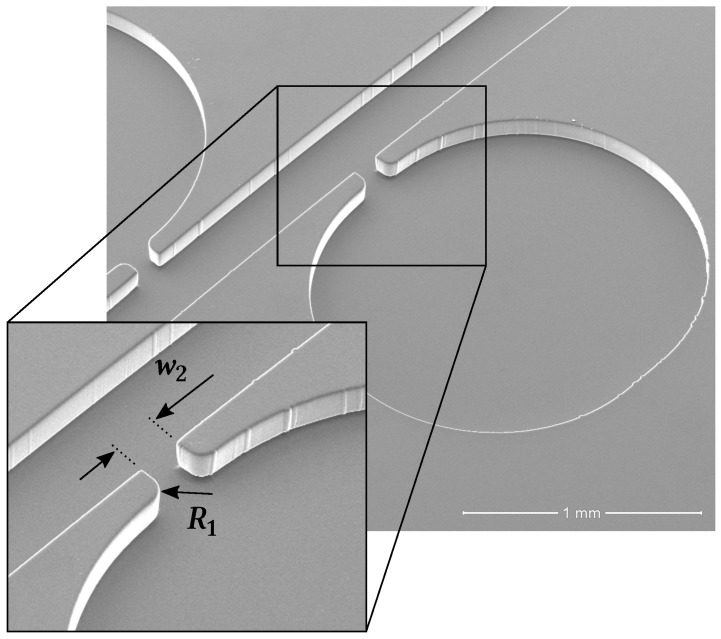
SEM image of the optimal pressure tap configuration: $w_{2}=108\;\mu m$ and $R_{1}=50\;\mu m$.

**Figure 7:**
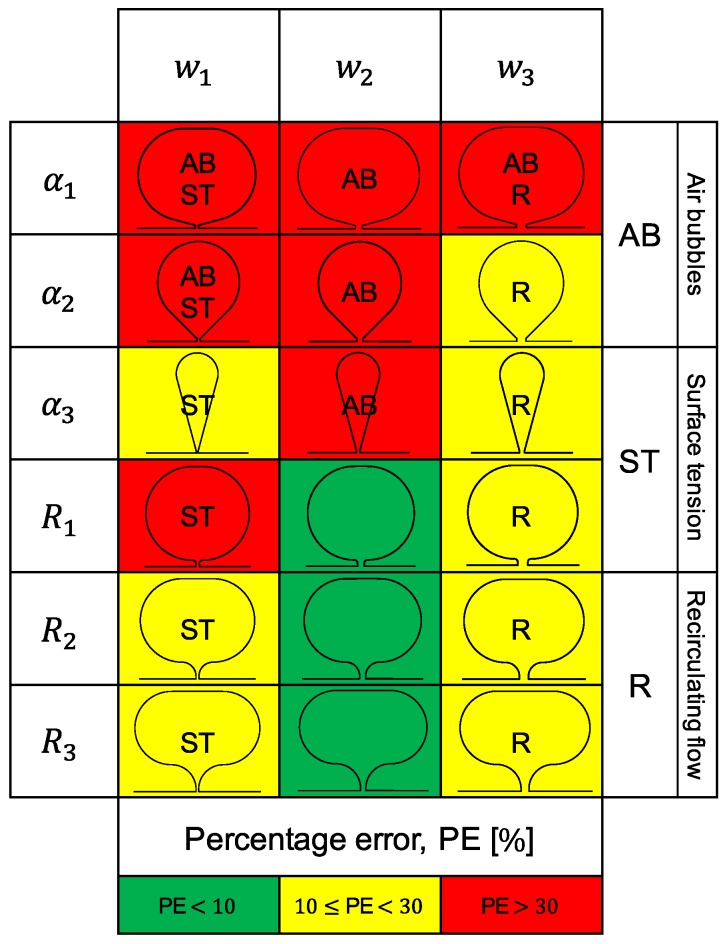
Summary of the results. The colour scheme reflects the average error in the experimental pressure drop, ΔP, when compared to the analytical solution, per pressure tap configuration.

The three $w_{2}$ tap configurations with smooth contours (*R*) displayed a very similar behaviour, having the results from all three approximately met the theoretical predictions. From Table 4 it can be concluded that the $R_{1}=50\;\mu m$ taps provided measurements with an average error below 5%. This configuration is then the optimal solution to our parametric study (see Figure 6). Nevertheless, all three $w_{2}$ taps are ‘optimal’ in the sense that they provide a means for measuring pressure with relative accuracy and little design and fabrication constrains. Our conclusions are valid for $0.2<w/2L_{c}<0.6$. Figure 7 summarises the results obtained. In this Figure, three key phenomena—air bubbles, surface tension and recirculating flow—were highlighted as the most probable causes for the distorted pressure measurements. For the pressure tap configurations that performed worst, the most likely of these effects to have caused the bad results were pointed out.

## 5. Conclusions

In this work, a total of 18 different pressure tap configurations were tested, using straight PDMS microfabricated channels. Pressure drop was measured along different length sections of the microchannels. The pressure gradient displayed a linear trend as expected. Out of the total number of pressure taps tested three stood out: those with an 108μm sub-channel width ($w_{2}$) and a smoothly shaped contour (*R* taps). Results obtained with these taps showed very good agreement with theory, scoring an average relative error of less than 10%. From Table 4 it can be concluded that the $R_{1}=50\;\mu m$ taps provided measurements with an average error below 5%, making them an optimal choice for pressure tap designs, at least for Newtonian fluids. Moreover, similar results are expected when working with viscoelastic fluids. These conclusions are true within the interval $0.2<w/2L_{c}<0.6$. Some minor non-linearity was detected in the results, yet only for Re≥50. This was likely caused by the compliance of the channels inner PDMS walls with increasing pressure. Since the pressure drop inside the microchannels was rather low, never exceeding ∼2.1kPa, the magnitude of this error was not worrying at lower Re. The results from sections AB, CD and AD were influenced by the built-in configuration of pressure taps A and D, ending up systematically far from the analytical profile. The configuration of these taps inherently adds expansion/contraction zones to the channel, which naturally disturb the flow. Data from section BC was then the most significant. Anomalous results from this section were linked to air bubbles housed inside the pressure taps, interfacial phenomena and geometrical imperfections related to the fabrication methods. The more or less likelihood for such effects to develop can be related to the shape of the taps. With that in mind, the following guidelines were written:

- Pressure taps with a very narrow sub-channel (w≤54μm) are likely to be affected by surface tension phenomena
- Shapes with edges or other similar geometrical features (α taps) perform worse than those with smooth contours (*R* taps)
- Pressure taps with a sub-channel width, *w*, of 108μm led to better results than those with a wider 162μm sub-channel width

## Figures and Tables

**Table 1:** Shape parameters considered for the 18 pressure tap configurations.

	$\alpha_{1}=15^{\circ }$	$\alpha_{2}=45^{\circ }$	$\alpha_{3}=75^{\circ }$	$R_{1}=50\;\mu m$	$R_{2}=250\;\mu m$	$R_{3}=500\;\mu m$
$w_{1}=54\;\mu m$	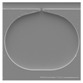	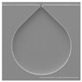	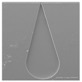	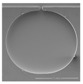	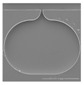	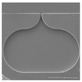
$w_{2}=108\;\mu m$	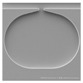	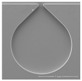	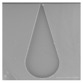	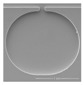	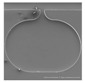	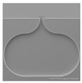
$w_{3}=162\;\mu m$	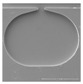	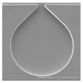	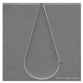	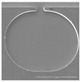	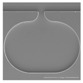	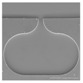

**Table 2:** Length, *L*, of the channel sections along which pressure drop measurements were performed.

	AB	BC	CD	AD
*L* (mm)	4.01	0.987	8.27	13.3

**Table 3:** Percentage error (%) between experimental and analytical data for pressure taps with sub-channel width $w_{1}=54\;\mu m$.

	Re					Re				
	$10^{-1}$	$10^{0}$	10	$10^{2}$		$10^{-1}$	$10^{0}$	10	$10^{2}$	Section
$\alpha_{1}$	–	80.0	73.3	77.0	$R_{1}$	–	73.3	82.2	82.6	AB
	–	68.2	55.3	63.0		–	80.0	53.1	76.6	BC
	70.4	76.9	70.7	–		100.0	83.3	80.9	–	CD
	82.5	86.1	74.8	–		83.2	89.1	83.2	–	AD
$\alpha_{2}$	–	80.5	81.0	72.5	$R_{2}$	–	69.8	88.6	82.2	AB
	–	31.2	61.6	62.2		–	12.8	66.0	71.7	BC
	80.0	72.1	73.7	–		71.9	86.6	72.5	–	CD
	93.8	74.6	71.1	–		64.6	79.0	79.3	–	AD
$\alpha_{3}$	–	76.0	77.8	74.3	$R_{3}$	–	74.3	81.2	76.8	AB
	–	138.0	25.5	62.9		–	38.4	20.2	76.3	BC
	32.2	48.6	64.9	–		26.8	83.1	77.3	–	CD
	91.2	71.7	69.8	–		64.0	76.6	78.8	–	AD

**Table 4:** Percentage error (%) between experimental and analytical data for pressure taps with sub-channel width $w_{2}=108\;\mu m$.

	Re					Re				
	$10^{-1}$	$10^{0}$	10	$10^{2}$		$10^{-1}$	$10^{0}$	10	$10^{2}$	Section
$\alpha_{1}$	–	52.3	51.6	–	$R_{1}$	–	52.6	58.4	52.4	AB
	–	91.7	31.1	39.0		–	3.3	3.3	8.5	BC
	89.9	62.0	59.7	–		4.50	45.1	57.0	–	CD
	54.8	63.0	59.8	–		46.5	60.9	55.9	–	AD
$\alpha_{2}$	–	95.1	65.6	–	$R_{2}$	–	32.5	54.7	–	AB
	–	92.5	62.9	61.9		–	13.2	1.9	16.6	BC
	38.1	64.0	62.5	–		31.7	45.2	45.5	–	CD
	74.8	43.1	58.2	–		44.0	44.2	48.2	–	AD
$\alpha_{3}$	–	43.0	58.0	63.3	$R_{3}$	–	–	–	–	AB
	–	40.5	47.0	56.2		–	6.5	8.0	31.2	BC
	14.9	62.3	65.3	–		–	–	–	–	CD
	49.4	15.3	65.7	–		–	–	–	–	AD

**Table 5:** Percentage error (%) between experimental and analytical data for pressure taps with sub-channel width $w_{3}=162\;\mu m$.

	Re					Re				
	$10^{-1}$	$10^{0}$	10	$10^{2}$		$10^{-1}$	$10^{0}$	10	$10^{2}$	Section
$\alpha_{1}$	–	69.3	86.0	74.7	$R_{1}$	–	71.5	57.8	53.9	AB
	–	73.3	71.5	79.8		–	23.9	32.6	35.6	BC
	50.5	78.1	74.6	–		15.2	35.6	42.1	–	CD
	59.8	81.9	77.4	–		24.6	15.8	32.9	–	AD
$\alpha_{2}$	–	64.4	79.6	67.0	$R_{2}$	–	58.2	64.3	–	AB
	–	53.9	25.2	56.8		–	38.9	16.1	30.2	BC
	72.4	71.4	64.1	–		26.6	45.1	43.2	–	CD
	70.4	63.9	68.8	–		55.1	41.4	36.6	–	AD
$\alpha_{3}$	–	63.7	79.7	65.6	$R_{3}$	–	56.2	64.6	57.0	AB
	–	26.7	64.8	59.6		–	35.6	15.9	40.8	BC
	64.2	55.0	56.2	–		41.1	35.0	52.9	–	CD
	18.8	66.6	56.7	–		28.7	35.9	42.8	–	AD

## Abbreviations

The following abbreviations are used in this manuscript:

DAQ	Data acquisition
DI	De-ionised
LOC	Lab-on-a-Chip
PAN	Polyacrylonitrile
PDMS	Polydimethylsiloxane
PE	Percentage error
Re	Reynolds number
SEM	Scanning electron microscopy
TMCS	Trimethylchlorosilane
